# Denaturing gradient gel electrophoresis of neonatal intestinal microbiota in relation to the development of asthma

**DOI:** 10.1186/1471-2180-11-68

**Published:** 2011-04-10

**Authors:** Carl Vael, Liesbeth Vanheirstraeten, Kristine N Desager, Herman Goossens

**Affiliations:** 1 Department of Microbiology, University of Antwerp, Antwerp, Belgium; 2 Department of Pediatrics, University of Antwerp, Antwerp, Belgium

**Keywords:** DGGE, infant, intestinal microbiota, asthma

## Abstract

**Background:**

The extended 'hygiene hypothesis' suggests that the initial composition of the infant gut microbiota is a key determinant in the development of atopic disease. Several studies have demonstrated that the microbiota of allergic and non-allergic infants are different even before the development of symptoms, with a critical time window during the first 6 months of life. The aim of the study was to investigate the association between early intestinal colonisation and the development of asthma in the first 3 years of life using DGGE (denaturing gradient gel electrophoresis).

**Methods:**

In a prospective birth cohort, 110 children were classified according to the API (Asthma Predictive Index). A positive index included wheezing during the first three years of life combined with eczema in the child in the first years of life or with a parental history of asthma. A fecal sample was taken at the age of 3 weeks and analysed with DGGE using universal and genus specific primers.

**Results:**

The Asthma Predictive Index was positive in 24/110 (22%) of the children. Using universal V3 primers a band corresponding to a *Clostridum coccoides XIVa *species was significantly associated with a positive API. A *Bacteroides fragilis *subgroup band was also significantly associated with a positive API. A final DGGE model, including both bands, allowed correct classification of 73% (80/110) of the cases.

**Conclusion:**

Fecal colonisation at age 3 weeks with either a *Bacteroides fragilis *subgroup or a *Clostridium coccoides *subcluster XIVa species is an early indicator of possible asthma later in life. These findings need to be confirmed in a new longitudinal follow-up study.

## Background

The increasing prevalence of asthma and other atopic diseases during the last decades was originally explained by the reduced exposure to infections early in life [1]. More recently Rautava et al.[2] suggested an extension of this "hygiene hypothesis" describing the importance of the initial composition of the infant gut microbiota as a key determinant in the development of atopic disease. This hypothesis is supported by studies demonstrating that the microbiota of allergic and non-allergic infants are different even before the development of symptoms, with a critical time window during the first 6 months of life [3]. The findings from these studies however are inconsistent: 4 different bacterial genera (*Staphylococcus, Bacteroides, Clostridium, Enterobacteriaceae*) are associated with an increased risk for atopic disease and 2 genera (*Bifidobacterium, Lactobacillus*) show a protective effect [4]. Most studies conducted so far were cross-sectional focusing on atopic dermatitis, only few studies considered asthma as outcome.

Until a decade ago, most of our knowledge on the composition of the intestinal microbiota was mainly based on culture dependent techniques. Comparisons with molecular methods have indicated that culture dependent methods underestimate intestinal microbiota diversity as only 10-50% of this population is culturable [5]. About 400 different species inhabit the human intestine based on culture methods, but using 16S rRNA sequencing more than 7000 different phylotypes were detected in the human gut [6]. Denaturing gradient gel electrophoresis (DGGE) is a molecular sequence dependent fingerprinting technique that allows to characterize the intestinal microbiota without pre-existing knowledge of its composition. DGGE using universal [7] and bifidobacterial primers [8] based on the bacterial 16S rRNA sequence has been applied successfully to monitor the development of the gut microbiota in infants.

In the Asthma and Allergy study we performed DGGE analysis of bacterial 16S rDNA genotypes on fecal samples to assess whether the intestinal microbiota of infants at the age of 3 weeks is associated with the development of asthma during the first 3 years of life.

## Methods

The Asthma and Allergy study is a prospective birth cohort and part of the Environmental Health action of the Flemish Ministry of Health and Environment. Children (n = 158) were recruited through maternity clinics in Flanders. Selection criteria for enrolment in the study were vaginal delivery at term and uncomplicated perinatal period. Questionnaires were collected with data on the parents, including demography, smoking and asthma. Data of the child on demography, respiratory symptoms and risk factors for asthma were collected by postal questionnaires sent every 6 months starting at the age of 3 weeks until the age of 36 months. The question on the presence of wheezing referred to the period between two questionnaires, e.g. the presence of wheezing in the questionnaire at 6 months referred to the time period between 3 weeks and 6 months. The study protocol was approved by the medical ethics committees of the participating institutes. All parents gave written informed consent.

Symptoms of wheeze were assessed by International Study of Asthma and Allergies in Childhood core questions [9]. Information about doctor's diagnosed parental asthma was collected by the following question: ''Did a doctor ever diagnose asthma?''. Based on the longitudinal questionnaire data on wheeze symptoms in the first 3 years of life, children were classified according to the 'loose' Asthma Predictive Index (API) into an API positive and an API negative group. According to the 'loose' index a positive API included wheezing during the first three years of life and eczema or parental history of asthma [10].

Approximately 2 g of stools was collected into a sterile recipient by the parents at 3 weeks of age. The sample was sent to the laboratory under anaerobic conditions where it was stored immediately at -70°C until analysis.

DNA was extracted from fecal samples based on the method of Pitcher et al. [11] modified by Vanhoutte et al. [5]. A saline suspension of feces was made by diluting 1 g of wet feces in 10 ml of sterile saline solution and homogenized using a stomacher. Of this fecal sample suspension, 1 ml was centrifuged at 20,000 *g *for 5 min. After removal of the supernatant, the pellet was resuspended in 1 ml TE buffer (10 mM Tris-HCl, 1 mM EDTA, pH 8.0) and was again centrifuged at 20,000 *g *for 5 min. The pellet was resuspended in 150 μl enzyme solution (6 mg lysozyme powder [Serva] and 40 μl mutanolysine [Sigma] dissolved in 110 μl TE (1 ×) per sample) followed by incubation at 37°C for 40 min. Next, 500 μl GES reagent (Guanidiumthiocyanate-EDTA-Sarkosyl; 600 g l^-1 ^guanidiumthiocyanate [Sigma], 200 ml l^-1 ^0.5 M EDTA, 10 g l^-1 ^sarkosyl) was added to complete all lysis, after which the solution was put on ice for 10 min. In the following step, 250 μl ammonium acetate (7.5 M) was added and the mixture was put on ice for 10 min. Subsequently, two chloroform-iso-amylalcohol extractions were performed with 500 μl chloroform/iso-amylalcohol solution (24/1). Finally, DNA was precipitated by adding 0.54 volumes of ice-cold isopropanol. After centrifugation at 20,000 *g *for 5 min, the pellet was washed twice with 150 μl 70% EtOH, air dried and allowed to dissolve overnight in 150 μl TE (1 ×) buffer. The remaining RNA was removed by adding 7.5 μl RNase (2 mg ml^-1^; Serva) after which samples were incubated for 1.5 h at 37°C. Purified DNA extracts were stored at -20°C.

PCR was performed with a *Taq *polymerase kit (Supertaq, HT Biotechnology Ltd). Each PCR mixture (50 μl) contained 6 μl 10 × PCR buffer (containing 15 mM MgCl_2_), 2.5 μl Bovine Serum Albumin (0.1 mg ml^-1^), 2.5 μl dNTP preparation (containing each dNTP at a concentration of 2 mM), 2 μl of each primer (5 μM); 0.25 μl *Taq *polymerase, 33.75 μl sterile Milli-Q water and 1 μl of 10-fold diluted DNA solution. One single PCR core program was used for all primer pairs: initial denaturation at 94°C for 5 min; 30 cycles of denaturation at 94°C for 20 s, annealing at primer-specific temperature (Table 1) for 45 s and extension at 72°C for 1 min; and final extension at 72°C for 7 min followed by cooling to 4°C. PCR amplicons were verified with electrophoresis in a 1.5% agarose gel after staining with ethidium bromide (50 μl in 500 ml 1 × TAE buffer [TE buffer with 5.71% (vol/vol) acetic acid]) with a 100-bp molecular ruler (Invitrogen) to compare with the expected amplicon size for the corresponding primer set (table 1) (data not shown). PCR amplification products were stored at -20°C.

**Table 1:** Specifications of the 16S rRNA primers used in this study

Target group (variable region)	Primer designation	Primer sequence (5'-3')	Amplicon size	Annealing temperature	DGGE gradient	Reference
Universal (V3)	F357-GC ^a^ 518R	TACGGGAGGCAGCAG ATTACCGCGGCTGCTGG	217	55°C	20-70%	Muyzer et al., 1993
Universal (V6-V8)	U968F-GC ^a^ L1401-R	AACGCGAAGAACCTTAC CGGTGTGTACAAGACCC	489	55°C	20-70%	Zoetendal et al., 1998
*Bacteroides fragilis *subgroup	Bfra 531F Bfra 766R-GC^a^	ATACGGAGGATCCGAGCGTTA CTGTTTGATACCCACACT	293	65°C	20-70%	Vanhoutte et al., 2006
*Bifidobacterium*	g-Bifid F g-Bifid R-GC^a^	CTCCTGGAAACGGGTGG GGTGTTCTTCCCGATATCTACA	596	65°C	40-70%	Matsukiu et al., 2002
*Lactobacillus *group^b^	Lac 1 Lac2-GC ^a^	AGCAGTAGGGAATCTTCCA ATTYCACCGCTACACATG	380	61°C	35-60%	Walter et al., 2001

^a ^Primers with GC clamp at 5' end: CGCCCGCCGCGCCCCGCGCCCGGCCCGCCGCCCCCGCCCC.

^b ^*Lactobacillus *group comprising the genera *Lactobacillus, Leuconostoc, Pediococcus *and *Weisella*.

16S rRNA gene amplicons were analyzed with DGGE as described previously [12]. In our study, different types of denaturing gradient were applied depending on the primers used (table 1). The polyacrylamide gels (160 by 160 by 1 mm) consisted of 8% (vol/vol) polyacrylamide (Biorad) in 1 × TAE buffer. By diluting a 100% denaturing polyacrylamide solution (containing 7 M urea [Biorad] and 40% formamide [Sigma]) with a polyacrylamide solution containing no denaturing components, polyacrylamide solutions with the desired denaturing percentages were obtained. The 24-ml gradient gels were cast by using a gradient former (Biorad) and a pump (Biorad) set at a constant speed of 5 ml/min. The denaturing gels were allowed to polymerize for 3 h, after which a 5-ml nondenaturing stacking gel containing a 16-well comb was poured on top. After 1 h of polymerization, PCR samples were loaded into the wells, and electrophoresis was performed for 16 h at 70 V in 1 × TAE buffer at a constant temperature of 60°C by using the Dcode system (Biorad). DGGE gels were stained for 30 min with 1 × SYBR^® ^Gold (Molecular Probes) in 1 × TAE buffer. This was followed by visualization of DGGE band profiles under UV light. Digital capturing was performed by using a Geldoc XR camera system (Biorad) combined with the Quantity One software package (Biorad). By including a standard reference every six lanes in each DGGE gel, it was possible to digitally normalize the gel profiles by comparison with a standard pattern using the BioNumerics software, version 2.5 (Applied Maths, St.-Martens-Latem, Belgium). This normalization enabled comparison between DGGE profiles from different gels provided that these were run under comparable denaturing and electrophoresis conditions.

The digitalized data were exported as an excel file for further statistical analysis according to Gafan et al. [13]. In this file, each sample represented 1 row and each band was assigned to a unique band position (column) with 1 indicating presence and 0 indicating absence of a band at that position. API was used as outcome parameter. The data were first analysed using Chi-square tests with a cut-off for significance at 5% to reduce the number of bands included in multivariate analysis. Significant bands (absent coded as 0, present coded as 1) were included as explanatory variables in a multivariate logistic regression analysis (API as dependent variable, negative coded as 0, positive as 1) for each primer set separately. The estimated odds ratio was calculated for each band in the logistic regression model. Bands remaining significantly associated with the API index in this model were adjusted for 5 known asthma confounders (exclusive breast feeding, maternal smoking during pregnancy, infant use of antibiotics at age of 3 weeks, parental socio-economic status and gender) in a second logistic regression model. The statistical analysis was conducted using SPSS version 15 (Chicago, USA).

Significant bands in this second model were identified after excision of the band from the gel and overnight incubation in TE buffer at 4°C. After extraction of the band it was reamplified with the corresponding primer set and reanalysed in DGGE together with the original fecal sample to confirm if the correct band was extracted. This process was repeated 2-3 times until a single band was obtained. This band was subsequently sequenced without additional cloning by the Genetic Service Facility (VIB, University of Antwerp) with a capillary sequencer (Applied Biosystems 3730 DNA analyser) using the corresponding forward and reverse primers without the GC clamp. In all cases this procedure resulted in a pure sequence product from the excised band. The obtained sequences were analysed using the BLAST program.

## Results

### Description of the study population

Of the 158 children recruited in this study, 54% were boys. Maternal or paternal asthma was present in 8% and 5% of the children, respectively. Several children were lost for follow-up at the end of the 3 year study period. As a result, API at age 3 years could not be determined in 41 of the 158 children due to missing data on wheezing (n = 30) or on eczema (n = 9) of the child in the 6 monthly questionnaires or on parental asthma (n = 5). As described previously, there were no differences in the percentage of children with wheeze at any age, parental asthma, and eczema at any age or gender of the infant between children who could or could not be categorized according to API [14]. In 7 children insufficient fecal sample was available to perform a DGGE analysis. API was positive in 24/110 (22%) of the remaining children.

### Fecal microorganisms in the study population

A total of 145 fecal samples were collected, which is a response rate of 92%.

The *Lactobacillus *and *Bifidobacterium *primers did not show any correlation with the API index (data not shown). With the universal V6-V8 primers only 1 single band (band 54.2) correlated significantly with the API index (Chi square, p = 0.04). After adjustment for exclusive breast feeding, maternal smoking during pregnancy, infant use of antibiotics at age of 3 weeks, parental socio-economic status and gender in a multivariate logistic regression analysis, the V6-V8 band 54.2 remained significantly associated with the API index (OR = 4.0, CI 1.2-12.9) (table 2). Excision and sequencing of band 54.2 revealed a DNA fragment of 397 bp [EMBL:FN611010] showing 98% similarity with an uncultured bacterial sequence isolated from a human fecal sample (table 3). The highest sequence similarity with a known species was obtained for *Eubacterium contortum, Clostridium oroticum *and *Ruminococcus torques *(table 3). These species belong to the *Clostridium *subcluster XIVa proposed by Collins et al. [15], which constitutes a major part of the human fecal flora [16].

**Table 2:** Multiple logistic regression analysis of risk factors for outcome variable Asthma Predictive Index at age 3 years

	V6-V8 band 54.2		V3 band 60.1		BF band 45.9		Final DGGE model*	
**Variable**	**OR**	**P**	**OR**	**P**	**OR**	**P**	**OR**	**P**

Presence of a DGGE band at the age of 3 weeks (yes versus no)	4,0	0,02	3,6	0,02	10,5	0,008	4,7	0,002
Exclusive breastfeeding age below the age of 3 weeks (yes versus no)	1,4	0,58	1,5	0,47	1,1	0,90	1,5	0,50
Maternal smoking during pregnancy (yes versus no)	1,4	0,69	1,7	0,56	1,6	0,58	1,9	0,47
Infant use of antibiotics below the age of 3 weeks (yes versus no)	0	1,0	0	1,0	0	1,0	0	1,0
Parental socioeconomic status (low versus high)	1,1	0,86	1,1	0,92	1,3	0,64	1,1	0,86
Infant gender (male versus female)	0,5	0,22	0,6	0,26	0,7	0,51	0,6	0,29

Odds ratios are adjusted for the independent variables, indicated in the rows of the table

OR: adjusted odds ratio

P: P value

BF: *Bacteroides fragilis *subgroup

* Final DGGE model: a combination of V3 band 60.1 (*Clostridium coccoides *subcluster XIVa) and *Bacteroides fragilis *subgroup band 45.9.

**Table 3:** BLAST identifications of the excised DGGE bands

DGGE amplicon			Identification		Sequence	# Bp	
**Band nr**	**Primer**	**# Bp**	**Species**	**Accession Nr**	**% identity**	**Identical**	**Total**

54.2	L1401-R (V6-V8)	397	*Uncultured bacterium*	EF405354.1	98	385	389
			*Eubacterium contortum*	L34615.1	93	364	390
			*Clostridium oroticum*	M59109.1	94	367	389
			*Ruminococcus torques*	L76604.1	93	365	389

60.1	518R (V3)	139	*Uncultured bacterium*	EF403112.1	100	124	124
			*Ruminococcus productus*	AY937379.1	98	122	124
			*Clostridium sp*.	Y10584.1	98	122	124
			*Ruminococcus hansenii*	M59114.1	97	121	124

45.9	Bfra 531F	241	*Bacteroides fragilis*	DQ100447.1	99	210	211
			*Bacteroides finegoldii*	AB222700.1	98	207	211
			*Bacteroides thetaiotaomicron*	AY319392.1	97	206	211

With the universal V3 primers only 2 bands (band 60.1 and 95.0) correlated significantly with the API index (Chi square, respectively p = 0.03 and p = 0.04). In a logistic regression analysis including both bands, only band 60.1 (OR = 5,9; CI 1,1 - 7,9) remained independently associated with the API index (band 95.0: OR = 5.7 10^9^; CI 0 - NA). After further adjustment for confounders in a multivariate logistic regression analysis, the V3 band 60.1 remained significantly associated with the API index (table 2). Excision and sequencing of band 60.1 revealed a DNA fragment of 139 bp [EMBL:FN611009] showing 100% similarity with an uncultured bacterial sequence isolated from a human fecal sample (table 3). The highest sequence similarity with a known species was obtained for *Ruminococcus productus or hansenii *and *Clostridium sp *(table 3). These species also belong to the *Clostridium *subcluster XIVa proposed by Collins et al. [15] with *Clostridium coccoides *as their nearest neighbour.

With the *Bacteroides fragilis *subgroup primers 4 bands (band 18.4; 27.3; 45.9 and 57.9) correlated significantly with the API index (Chi square, respectively p = 0.008; 0.048; 0.006 and 0.048). In a logistic regression analysis including all 4 bands only band 45.9 (OR = 7.1; CI 1,1 - 46,1) remained independently associated with the API index (band 18.4: OR = 4,8; CI 0,3 - 80,0/band 27.3: OR = 8,6 10^7^; CI 0 - NA/band 57.9: OR = 8,6 10^7^; CI 0 - NA). After adjustment for confounders, the *Bacteroides fragilis *subgroup band 45.9 remained significantly associated with the API index (table 2). Excision and sequencing of band 45.9 revealed a DNA fragment of 241 bp [EMBL:FN611011] showing 99% similarity with *Bacteroides fragilis *(table 3). A similarity of 98 and 97% was found with respectively *Bacteroides finegoldii *and *Bacteroides thetaiotaomicron *(table 3). In a final logistic regression model including the 3 significant DGGE bands only V3 band 60.1 (OR = 3,4; CI 1,2 - 9,7) and the *Bacteroides fragilis *subgroup band 45.9 (OR = 9,8; CI 1,6 - 59,3) proved to be independent variables excluding the V6-V8 band 54.2 (OR = 2,3; CI 0,7 - 7,8) from the model. After adjustment for confounders, this simple final DGGE model including only 2 bands (band 60.1 and band 45.9) remained significantly associated with the API index (table 2). The accuracy of predicting asthma at the age of 3 years using this final DGGE model is shown in table 4. The model allows correct classification of 73% (80/110) of the cases.

**Table 4:** Accuracy of final DGGE model* in predicting API status at age 3 years

	API index		N	
	Pos	Neg		
DGGE model Pos.	13	19	32	PPV = 41%
DGGE model Neg.	11	67	78	NPV = 86%
Total	24	86	110	

	54% S	78% Sp		X^2^, p = 0.002

Overall correct classification: 80/110 = 73%

API prevalence: 24/110 = 22%

Final DGGE model:

Positive: presence of band 60.1 (*Clostridium coccoides *subcluster XIVa) or band 45.9 (*Bacteroides fragilis *subgroup)

Negative: absence of band 60.1 (*Clostridium coccoides *subcluster XIVa) and band 45.9 (*Bacteroides fragilis *subgroup)

N: number of cases

PPV: positive predictive value

NPV: negative predictive value

S: sensitivity

Sp: specificity

This means that, according to our findings, early intestinal colonization of infants with bacteria belonging to the *Bacteroides fragilis *group and/or to the *Clostridium coccoides *subcluster XIVa is associated with an increased risk for the development of asthma at the age of 3 years. These bacteria are strict anaerobes and are part of the dominant genera of the normal intestinal microbiota observed in adults. We could not detect any bacterial taxa that were associated with health (API negative status). *Lactobacillus *and *Bifidobacterium*, the bacterial genera generally used as probiotics and considered by definition of having a beneficial effect on health could not be associated with a reduced risk of asthma. However it cannot be excluded that our inability to demonstrate a beneficial effect of certain bacterial taxa on infant health was caused by the limited sensitivity of the DGGE method that we used.

## Discussion

This study shows an association between early colonisation with a *Bacteroides fragilis *subgroup species and asthma later in life. We also showed in this study that a *Clostridium coccoides *subcluster XIVa species is an early indicator of asthma later in life. This is the first prospective study that links *Clostridium coccoides *subcluster XIVa to API, a clinically relevant risk factor for developing asthma. Differences in feeding pattern, use of antibiotics, gender, maternal smoking in pregnancy or parental socio-economic status cannot explain the findings.

Asthma is a frequently occurring condition in children with up to 50% of infants and children suffering of one or more episodes of wheezing below the age of 6 years. The diagnosis of asthma is not straightforward since no simple clinical tools are available to discriminate children prone to develop persistent asthma from those who will not. The 'Asthma Prediction Index' has been associated with an increased risk for asthma at school age [10]. This index was chosen as outcome parameter for the study since it is at present the best tool for prediction of asthma at school age. We used the so-called 'loose' index, which only required infrequent wheezing episodes in early life combined with risk factors for asthma because it has a much higher sensitivity (39%) but slightly lower specificity (82%) and positive predictive value (32%) than the so-called "stringent" index. The negative predictive value at all ages was very high for both indices, suggesting that the great majority of children who did not develop asthma during the school years had a negative predicted index during the first years of life. Because the Asthma Predictive Index is only an approximation to predict which children will subsequently develop persistent asthma, further follow-up at school age is required to definitely determine the relation between early *Bacteroides fragilis *and *Clostridium coccoides *subcluster XIVa colonisation and asthma.

With the exception of our previous study [14] using conventional culture methods, there are no data linking the *Bacteroides fragilis *subgroup to asthma but several studies showed a correlation between *Bacteroides *and allergy: A higher IgG immune response to *Bacteroides vulgaris *was found in high school children with allergic symptoms [17]. A positive correlation between the fecal counts of *Bacteroides *and the serum IgE concentration was demonstrated in 2 studies, one in infants intolerant to an extensively hydrolysed formula [18] and one in non-allergic children at the age of 5 years [19]. A study in adults with pollen allergy showed an increased ratio of fecal counts of *Bacteroides fragilis *to *Bifidobacterium *during pollen season. In vitro, using peripheral blood mononuclear cells of these patients, they also demonstrated that *Bacteroides fragilis *strains induced more Th2 cytokines but fewer Th1 cytokines compared with *Bifidobacterium *strains [20]. We believe that intestinal *Bacteroides *species might be able to induce a Th2 cytokine response through binding of a TLR2 (Toll-like receptor) present on intestinal dendritic cells. Netea et al. showed that *Bacteroides *species stimulate cytokine release through TLR2-dependent (not TLR4) mechanisms [21]. TLR2 agonists induce a Th2 response by suppressing IL-12 production [22].

Fecal *Clostridium *colonisation in infants has been linked to asthma before: A higher level of *C. difficile*-specific IgG was found in one-year-old children with recurrent wheezing [23]. A higher prevalence of *C. difficile *was detected using quantitative real-time PCR in infants who developed recurrent wheeze during the first 2 years of life [24]. *C. difficile *belongs to *Clostridium *cluster XI and is only remotely related to the *Clostridium coccoides *subcluster XIVa species that we detected [15]. These differences might be explained by the use of different methods (DGGE versus quantitative PCR or serology) and because different clinical outcomes were determined (API at the age of 3 years versus recurrent wheezing at the age of 1 or 2 years). In adult fecal microbiota *Clostridium coccoides *subcluster XIVa is the most abundant taxonomic group [16] but in infants it normally constitutes only a subdominant group at much lower counts [25]. Through the peptidoglycan present in their bacterial cell membrane the intestinal *Clostridium *species might be able to induce a Th2 cytokine response by binding to the TLR2 of the intestinal dendritic cells [21,22].

Several studies used DGGE to examine the relationship between the composition of the intestinal microbiota and the development of allergy and eczema [26-28]. In a case-control study, the prevalence of one specific DGGE band (identified as *E. coli*) was higher in infants with eczema [26]. A reduced fecal microbial diversity was observed with DGGE in allergic children [27] or in infants with eczema [28]. Only one study looked at wheezing as outcome using DGGE but did not find a difference in gut microbiota between wheezing and non-wheezing children at the age of 3-5 years [29]. We found a difference in the composition of the fecal microbiota at the age of 3 weeks but not later (at the age of 6 and 12 months; data not shown), illustrating the importance of a critical time window during the first 6 months of life [3].

Prematurity is a much more complex situation than the normal population observed in our study. A delay of up to 6 months of the intestinal *Bacteroides *colonization, which occurs in newborns after caesarean section, might even decrease the subsequent risk for asthma in these premature infants according to our findings. However, genetic factors or the underlying disease that provoked the premature delivery itself might significantly increase the subsequent risk for asthma. Future studies on premature newborns and their respiratory disease outcome should not only include the intestinal microbiota but should also correct for confounders like antibiotic use, mode of delivery and underlying disease or genetic mutations.

Despite obvious advantages, DGGE also has a limitation. The detection limit of DGGE is estimated to approach 1% of the total population or a concentration of 10^6 ^CFU/g feces. This is significantly higher than the detection limit of the culture method we used in our previous study (detection limit ≥ 10^3 ^CFU/g feces) [14]. Another limitation of the present study is the fact that no stool sample of the mother was included, so we cannot make any statement on the origin of the *Bacteroides *and *Clostridium *strains recovered in these infants. Finally, as in every longitudinal study, missing data is a problem. Since there were no differences in the percentage of children with wheezing, eczema or parental asthma or gender of the infant between children who could or could not be categorized according to API, it seems improbable that the missing data resulted in a systemic bias. Adjustment for known confounders in a multivariate logistic regression analysis further confirmed our findings.

## Conclusions

Fecal colonisation at age 3 weeks with either a *Bacteroides fragilis *subgroup or a *Clostridium coccoides *subcluster XIVa species is an early indicator of possible asthma later in life. These findings need to be confirmed in a new longitudinal follow-up study. The effect of pre- and probiotics on the intestinal colonization with *Clostridium *and *Bacteroides *requires further attention in future trials for the prevention of asthma in infants and children.

## Abbreviations

API: Asthma Predictive Index; CI: confidence interval; DGGE: denaturing gradient gel electrophoresis; TLR: Toll-like receptor

## Competing interests

The authors declare that they have no competing interests.

## Authors' contributions

CV was involved in the study design and concept, helped to draft and revise the manuscript and performed the statistical analysis. LV assisted in the data acquisition and helped revising the manuscript. HG was involved in the study design and concept and helped revising the manuscript. KD was involved in the study design and concept and helped to revise the manuscript. All authors read and approved the final manuscript.
